# Comparison of toxic heavy metals in the breast milk of diabetic and non-diabetic postpartum mothers in Yenagoa, Nigeria

**DOI:** 10.1371/journal.pone.0264658

**Published:** 2023-04-07

**Authors:** Tuboseiyefah Perekebi Philip-Slaboh, Chinemerem Eleke, Anthonet Ndidiamaka Ezejiofor

**Affiliations:** 1 African Centre of Public Health and Toxicological Research (ACE-PUTOR), University of Port Harcourt, Port Harcourt, Nigeria; 2 Department of Nursing Science, University of Port Harcourt, Port Harcourt, Nigeria; Zagazig University, EGYPT

## Abstract

**Background:**

Breast milk is the main source of neonatal nutrition. It is not known whether diabetes increases the excretion of toxic heavy metals in the breast milk of postpartum mothers. We compared the concentration of toxic heavy metals in breast milk between diabetic and non-diabetic postpartum mothers in Yenagoa.

**Material and methods:**

A cross-sectional design was utilized on a purposive sample of 144 consenting postpartum mothers (72 diabetic and 72 non-diabetic mothers) from three public hospitals. Breast milk samples were collected at 5–6 weeks postpartum between 1st November 2020 and 30th April 2021. Atomic-Absorption-Spectrophotometer and Direct-Mercury-Analyzer were used to analyze the breast milk samples. A data collection form (proforma) was used and data were analyzed at a 5% significance level with IBM-SPSS 25 software.

**Result:**

High levels of Arsenic (63.9% vs. 62.5%), Lead (95.8% vs. 95.8%), Mercury (68.1% vs. 72.2%), and Cadmium (84.7% vs. 86.1%) were detected in the breast milk of the diabetic and non-diabetic groups respectively. The mean concentrations for Arsenic (0.6 vs. 0.6 ng/mL), Lead (13.2 vs. 12.2 ng/mL), Mercury (2.9 vs. 3.0 ng/mL), and Cadmium (3.3 vs. 3.2 ng/mL) were above the WHO permissible limits, thus showing evidence of risk to the health of the mother and neonate. There was no significant difference in the concentration of toxic heavy metals in breast milk between the groups (p = > 0.585).

**Conclusions:**

Diabetes did not seem to increase the concentration of toxic heavy metals expressed in breast milk. More rigorous studies are needed to confirm these findings.

## Introduction

Typically, the postpartum period begins soon after birth and lasts approximately six weeks. Within this period, maternal breast milk is a source of nutrition to the newborn. Normal breast milk contains about 87% of water, 7% of carbohydrates, 4% of lipids, and 1% of proteins, vitamins, and minerals [1]. A disease such as diabetes could alter the electrolyte composition of the human body through the production of abnormally large amounts of urine [2]. The resulting dehydration and electrolyte deficiency could trigger adaptive mechanisms that could mobilize ingested heavy metals into breast milk [2]. The presence of heavy metals in breast milk does present toxicological fears concerning neonatal health [3]. In line with this, the concentration of toxic heavy metals in breast milk requires monitoring especially in regions where environmental pollution is apparent.

Environmental pollution is linked with one in every six global deaths, and this amounts to about nine million deaths annually [4]. In 2015, polluted air accounted for roughly six million deaths globally [5]. In the same year, water pollution contributed approximately two million deaths [6]. Unfortunately, 92% percent of pollution-related deaths occur in low and middle-income countries of the world [4]. Since the growth of global industrialization and fossil fuel exploration, toxic heavy metals such as Arsenic (As), Lead (Pb), Mercury (Hg), and Cadmium (Cd) have progressively been detected in the environment. They make their way into the air and water from petrochemical factories waste, crude-oil refinery emissions, agriculture and mining industry effluents [7]. Some researchers have implied that when humans are exposed to environments contaminated with high concentrations of heavy metals, these metals can accumulate in human tissue over time [1, 2, 7]. Infants who depend largely on the postpartum mothers’ breast milk may also be at risk of ingesting the named toxic heavy metals from their mothers [3, 8].

Heavy metals exposure could trigger suppression of cytokine production and lower immunity as they bind to albumin and enter the breast milk [8]. Toxicity from arsenic is currently a public health concern [9]. When arsenic is ingested, it causes skin hypo-pigmentation, peripheral vascular diseases such as black foot disease, gangrene, and high blood pressure [10]. In pregnant women, the chance of cancer is increased when arsenic is ingested and it stimulates growth delay in fetuses. Its presence as a contaminant in breast milk has also been reported [11]. Lead is a heavy metal that naturally exists in rocks and present in leaded gasoline. It has no known nutritional value. Contaminated food, water, and air are all sources of lead exposure in humans [12]. In this regard, residential locations near waste yards may have contaminated air, soil, and water. Previous data indicate that when calcium levels in breast milk are low, lead stored in a woman’s bone marrow is physiologically mobilized to match the need [2]. Furthermore, the half-life of lead in human blood is 30 days, whereas the half-life of lead in bone is 27 years. Based on the data, it appears that a breastfeeding mother will continue to shed lead for several days after the initial exposure. Mercury is a heavy element that occurs naturally in the earth’s crust. It is frequently emitted into the environment as a fume by insecticides, herbicides, and battery leaks [13]. Evidence has shown that fish accumulates up to 15 folds more mercury than lead [2]. In line with the mentioned, mercury is ultimately ingested by fish eaters. Cadmium enters the soil and running water through the improper disposal of sewage and computer waste [14]. Literature shows that high quantities of cadmium have been found in oysters, crabs, and the liver and kidneys of some farm animals [12]. Cadmium accumulates in the muscles over a lifetime in humans due to its delayed renal clearance and a half-life of up to 38 years. Evidence suggests that iron deficiency is linked with higher intestinal absorption of cadmium [2]. The postpartum period as a period of low iron stores puts the breastfeeding mother at even greater risk [2, 3].

Yenagoa is a Seaside town with staple consumption of charcoal smoked fish, heavy agricultural fertilizer use, and widespread clandestine (illegal) crude oil refining. It is also the seat of legal crude oil exploration and petrochemical activities in the Niger Delta region. It has experienced several crude oil spills from 1976 till recent times resulting in environmental degradation and the possible release of heavy metals into the environment [13]. The research team wonders if maternal diabetes would worsen the excretion of toxic heavy metals in breast milk. A literature search in the major research databases such as MEDLINE, PubMed, and ProQuest yielded few peer-reviewed publications on the subject matter. The knowledge gap motivated the research team to embark on a study to compare the concentration of heavy metals in the breast milk of diabetic and non-diabetic postpartum mothers in Yenagoa, Bayelsa State, Nigeria. To the best of our knowledge, this study is the first to measure heavy metals in the breast milk of diabetic lactating mothers.

## Material and methods

### Ethics statement

This study was approved by the University of Port Harcourt Institutional Review Board (Approval ID: UPH/CEREMAD/REC/MM75/050) and adhered to the Helsinki Declaration of 1975, as revised in 1983. All participants in this study gave their written consent. Participants younger than 18 years old provided consent through one of their parents.

### Study area and period

This study was conducted in the postnatal clinics of three randomly selected public hospitals. The hospitals were Federal Medical Centre, Diete-Koki Memorial Hospital, and Women Affairs Clinic, all in Yenagoa catchment area. Yenagoa is the capital of Bayelsa State and is located deep in the Niger Delta region, in the southern part of Nigeria. It lies between Latitude 4°15^’^ North and Latitude 5°23’ South, and between Longitude 5°22^’^ West and 6°45’ East. Yenagoa has commercial quantities of crude oil and about 700,000 residents whose major occupation is fishing and farming. The crude oil exploration activities by multinational companies alongside some clandestine crude oil refining by unlicensed persons make Yenagoa prone to environmental pollution from crude oil and factory waste [15, 16]. The consequent pollution of air, soil, and water raises the potential for human ingestion of toxic heavy metals.

### Study design and population

A cross-sectional design was employed for this study. The study population was an estimated 1,843 postpartum mothers aged between 15 and 49 years. This estimated figure was arrived at by summing up all registered postpartum mothers who obtained postnatal services from the facilities in the year 2019.

### Sample size determination and sampling procedure

A sample size of 144 postpartum mothers (72 diabetic and 72 non-diabetic mothers) was determined for this study using the Cochran’s formula mathematically stated as n = Z^2^ P(1-P)÷d^2^ [17]; where n = minimum sample size; Z = critical value constant 1.96; P = fertility rate for Bayelsa State 4.4% [18]; d = error level 5%. A minimum sample size of 65 was computed. The minimum sample size was increased by 10% using the non-response formula mathematically stated as n* = n ÷ (1–0.1) [19]; where n* = final sample size and n = minimum sample size of 65. A final sample size of 72 was computed for each of the two arms of the study.

The purposive sampling technique was applied to the selection of consenting participants. The inclusion criteria involved being a nursing mother and at 5–6 weeks postpartum period. This time period was chosen as postpartum mothers attend hospitals and clinics for their first routine medical check-up at about 5–6 weeks. Mothers who were resident outside Yenegoa or reported other co-morbidities outside diabetes were excluded from the study.

### Instrument and data collection

#### Questionnaire

A novel nine-item semi-structured data extraction form (proforma) with an inter-rater reliability of 0.938 was used for this study. It had two sections (A and B). Section A was designed to extract the background demographic characteristics of the participants such as age, marital, parity, employment, and disease status. Section B was designed to extract the result of breast milk laboratory analysis for toxic heavy metal composition and concentration.

#### Breast milk collection

Data and breast milk sample collection took place between November 2020 and April 2021. The purpose of this study was explained to the selected participants. The research team encouraged the consenting participants to give data on their background characteristics using the proforma (section A). For the collection of breast milk samples, 144 acid-washed 10 mL specimen collection plastic bottles were used. Nitric acid (10% strength) was used for washing the specimen bottles, after which they were rinsed with distilled water. A unique number was coded onto each proforma and matched with the labels on the acid-washed specimen collection bottles. The participants were encouraged to manually squeeze 10 mL of breast milk into the specimen collection bottles and the bottles were put into a small size plastic cooler, packed with ice, and transported for further laboratory analysis in the chemistry laboratory of Ohemerge Company Limited in Port Harcourt, Rivers State. The breast milk samples were tested for heavy metals by the Laboratory Scientist using Atomic Absorption Spectrophotometry.

#### Laboratory analysis

Each of the specimen bottles was washed with 9 mL Nitric acid (10% strength), after which they were rinsed with 10 mL distilled water. A 2ml aliquot of breast milk was taken from each sample bottle and digested with 5 mL of Nitric acid (65% strength) in an ETHOS UP microwave digestion machine (Milestone Inc, Shelton, USA) for 60 minutes at 90°C. To make the digest up to 15ml, distilled water was added to it. Blank (deionized water) samples were prepared using the same process. The detection and quantification of the toxic heavy metals were done in an Atomic Absorption Spectrophotometer (GFAAS, the PinAAcle 900AA Perkin Elmer model) with a graphite furnace and hollow cathode lamp operated at 283.3 and 228.8 nm wavelengths and current of 5 and 6 mA for Lead and Cadmium respectively. For Arsenic, the GFAAS machine was operated at 193.7 nm wavelength and current at 8 mA. A bandwidth of 0.7 nm was set on the machine. The detection limit for the GFAAS machines was 1 ng/mL. A three-point calibration in a linear range was performed for Arsenic (2, 4, and 8 ng/mL), Cadmium (2, 4, and 8 ng/mL) and Lead (5, 15, and 25 ng/mL). Each cycle of analysis always started with the analysis of a blank and each batch of tests ended with a method correction blank analysis. For mercury detection, the Direct Mercury Analyzer (DMAS 80A, Perkin Elmer) with thermal decomposition atomic absorption capability was used without any further sample preparation. The detection limit for the DMAS 80A machine was 0.005 ng/mL. Each sample from one study participant was tested 2 times and an average was computed and recorded into the data extraction proforma (section B of the questionnaire) by the certified laboratory scientist.

### Data analysis

Participants’ demographic data were summarized using descriptive statistical methods such as frequency and percentage. Interval data from age were summarized using mean, standard deviation, frequency, and percentage. Test of statistical difference between groups was tested using the t-test, Chi-square, and Fisher exact test inferential statistics at a 5% level of significance. All statistical analysis was done with the aid of Statistical Products and Service Solutions software version 25 (IBM Chicago IL, USA).

## Result

Table 1 summarizes the background characteristics of the participants and it revealed no significant difference between the diabetes and non diabetes groups in terms of Age (p = 0.418), parity (p = 0.738), marital status (p = 0.120), and employment (p = 0.405). Table 2 summarizes the concentrations of toxic heavy metals in the breast milk of diabetic postpartum mothers and revealed that the majority of the participants in the Diabetic group had high levels of Arsenic (63.9%), Lead (95.8%), Mercury (68.1%), and Cadmium (84.7), above the WHO permissible limits. The mean concentrations compared with the WHO permissible limits of the toxic heavy metals in the sample were Arsenic (0.6 vs. 0.0 ng/ml), Lead (13.2 vs. 5.0 ng/ml), Mercury (2.9 vs. 1.7 ng/ml) and Cadmium (3.3 vs. 1.0 ng/ml). Table 3 summarizes the concentration of toxic heavy metals in the breast milk of the Non-diabetic group and revealed that the majority of the participants had high levels of Arsenic (62.5%), Lead (95.8%), Mercury (72.2%), and Cadmium (86.1), above the WHO permissible limits. The mean concentrations compared with the WHO permissible limits of the toxic heavy metals in the sample were Arsenic (0.6 vs. 0.0 ng/ml), Lead (12.2 vs. 5.0 ng/ml), Mercury (3.0 vs. 1.7 ng/ml), and Cadmium (3.2 vs. 1.0 ng/ml). Table 4 compares toxic heavy metal concentrations in the breast milk of Diabetic and Non-diabetic groups and revealed no significant difference between the groups (p > 0.05).

**Table 1:** Background characteristics of the participants N = 144.

Category	Non-Diabetic group n (%)	Diabetic group n (%)	χ^2^	p value
**n**	72	72		
**Age (in years)**			6.04	0.418
15–19	10 (13.9)	11 (15.3)		
20–24	6 (8.3)	6 (8.3)		
25–29	10 (13.9)	14 (19.4)		
30–34	8 (11.1)	11 (15.3)		
35–39	18 (25.0)	11 (15.3)		
40–44	15 (20.8)	9 (12.5)		
45–49	5 (6.9)	10 (13.9)		
**Marital status**			2.42	0.120
Single	22 (30.6)	31 (43.1)		
Married	50 (69.4)	41 (56.9)		
**Parity status**			0.11	0.738
Primipara	35 (48.6)	35 (48.6)		
Multipara	37 (51.4)	37 (51.4)		
**Employment status**			0.69	0.405
Unemployed	39 (54.2)	39 (54.2)		
Employed	33 (45.8)	33 (45.8)		

n = frequency, % = percent, **χ**^**2**^ = chi square, p < 0.05 is significant

**Table 2:** Concentration of toxic heavy metals in the breast milk of the diabetic group N = 72.

Parameters	Arsenic	Lead	Mercury	Cadmium
Below WHO limits n (%)	26 (36.1)	3 (4.2)	23 (31.9)	11 (15.3)
Above WHO limits level n (%)	46 (63.9)	69 (95.8)	49 (68.1)	61 (84.7)
Mean (SD) ng/ml	0.63 (0.36)	13.20 (5.13)	2.98 (1.91)	3.32 (1.86)
Minimum ng/ml	0.0	4.3	0.0	0.0
Maximum ng/ml	1.2	22.4	6.3	6.3
WHO limits ng/ml	0.0	5.0	1.7	1.0

n = frequency, % = percent, SD = standard deviation, ng/ml = nanogram per mililitre

**Table 3:** Concentration of toxic heavy metals in the breast milk of the non-diabetic group N = 72.

Parameters	Arsenic	Lead	Mercury	Cadmium
Below WHO limits n (%)	27 (37.5)	3 (4.2)	20 (27.8)	10 (13.9)
Above WHO limits n (%)	45 (62.5)	69 (95.8)	52 (72.2)	62 (86.1)
Mean(SD) ng/ml	0.65 (0.36)	12.24 (5.13)	3.08 (1.74)	3.24 (1.87)
Minimum ng/ml	0.0	3.8	0.0	0.0
Maximum ng/ml	1.2	22.3	6.3	6.3
WHO limits ng/ml	0.0	5.0	1.7	1.0

n = frequency, % = percent, SD = standard deviation, ng/ml = nanogram per mililitre

**Table 4:** Comparison of toxic heavy metal concentrations in the breast milk of diabetic and non-diabetic groups N = 144.

Variable	Diabetes group n	Non-diabetic group n	t-test	χ^2^	P value
**Arsenic** Mean(SD)	0.63 (0.36)	0.65 (0.36)	0.39		0.696
Below limits	26	27		0.03	0.863
Above limits	46	45			
**Lead** Mean(SD)	13.20 (5.14)	12.24 (5.13)	-1.12		0.263
Below limits	3	3		0.00	1.000
Above limits	69	69			
**Mercury** Mean(SD)	2.90 (1.91)	3.08 (1.74)	0.31		0.757
Below limits	23	20		0.30	0.585
Above limits	49	52			
**Cadmium** Mean(SD)	3.32 (1.86)	3.24 (1.87)	-0.25		0.803
Below limits	11	10		0.06	0.813
Above limits	61	62			

n = frequency, SD = standard deviation, Mean(SD) was meaaured in ng/ml, p < 0.05 is significant

## Discussion

This study found that majority of the participants in the Diabetes group had high concentrations of Arsenic, Lead, Mercury, and Cadmium in their breast milk (higher than the WHO permissible limits). The implication of this finding is that breastfeeding infants of majority of the examined participants may be at risk of mental retardation [8]. Given that this study is the first to measure the heavy metals in the breast milk of diabetic women, there were no previous studies for direct comparison. Nonetheless, this finding is similar to reported findings involving non-diabetic mothers. For example, this finding is partially similar to reported findings in a study in Egypt that found that Lead (2,920 vs. 5.0 ng/ml) and Cadmium (16800 vs. 1.0 ng/ml) at toxic concentrations higher than the WHO permissible limits, but did not detect Arsenic and Mercury in the breast milk samples in Damietta [20]. Moreover, the concentrations of Lead and Cadmium that were found in the study in Egypt were about 200 folds more for Lead and 500 folds more for Cadmium. The dissimilarity in findings is connected to the activities predominant in the area of study. Diametta was reported to be an agrarian society that uses compost as fertilizers and has a high factory and vehicular traffic emissions. Compost is known to increase the soil composition of Copper, Zinc, and Lead. Additionally, petrol and gas cars are likely to librate even more Lead while factories will emit cadmium in exhaust fumes. In contrast, the area of this present study is not known for its use of compost as fertilizer but crops have been reportedly destroyed over the years due to environmental crude oil spills [15]. Due to the disparity in the qualities of the studied areas, the dissimilarity in findings between the studies was expected. Nonethless, this finding was lower than that reported in a study conducted in Cyprus that found Arsenic (730 ng/ml vs. 0.0 ng/ml), Lead (1,190 vs. 5.0ng/ml), and Cadmium (450 vs. 1.0 ng/ml) at very high amounts in breast milk samples. The discrepancy in findings could be linked to the inequalities in sample attributes utilized in the study [21]. The study in Cyprus examined breast milk samples that were drawn from a mix of urban and rural postpartum women hence a heterogeneous sample. A heterogeneous sample limits the internal validity of the study. In contrast, this present study examined breast milk samples drawn from a homogenous urban population. In the light of the fore mentioned, the findings between this study and the study in Cyprius were expected to differ.

This study found that the majority of the participants in the Non-diabetic group had high levels of Arsenic, Lead, Mercury, and Cadmium in their breast milk samples (above the WHO permissible limits). This finding would suggest that the immune system of the infants of majority of the examined participants could be under threat of suppression [8]. This finding corroborates a study in Zanziba which reported that Lead (707 vs.5.0 ng/ml) and Cadmium (311 vs. 1 ng/ml) were found in breast milk samples at levels that were greater than the WHO set limits [22]. Likewise, the Lead and Cadmium levels reported in the study in Zanziba were greater than what was found in this study by 25 and 100 folds respectively. The dissimilarity in findings is connected to the points in the postpartum period when the breast milk samples were collected for examination. The study in Zanziba collected breast milk samples from participants irrespective of the number of weeks into the postpartum period that the participants were. Based on the context that the excretion of toxic heavy metals is more expressed in the colostrum than in mature milk, a mix of breast milk samples at different stages of lactation limits the internal validity of the study in Zanziba [3]. Breast milk samples obtained from women at more than 6 weeks postpartum were likely to provide findings that were not a fair representation of the snapshot of the phenomena. We collected breast-milk samples at 5–6 weeks into the postpartum period. It was done to provide a more valid snapshot of the concentrations of the toxic heavy metals at one specific point in time across the sample examined. The dissimilarity in findings between this study and the study in Zanziba was hence expected. Likewise, this finding did not completely support another study in Iran that reported that Lead (7.15 vs. 5.0 ng/ml) and Cadmium (1.07 vs. 1 ng/ml) were detected in breast milk and Lead was notably above the WHO preset limit [23]. The similarity in findings could be connected to the design utilized in the study. Both the study in Iran and the present study utilized the cross-sectional design. However, the levels reported in the study in Iran were lower than the values noted in this present study. The discrepancy in value could be linked to the method of laboratory examination of the breast milk samples utilized. The study in Iran utilized the anodic stripping voltammetry method, whereas this present study utilized the Atomic Absorption Spectrophotometry method. Inherent disparity in processing and analysis between the two methods may have resulted in the discrepancy in concentrations.

This study found no statistically significant difference in the level of toxic heavy metals in breast milk between the Diabetes and Non diabetes groups. This finding may imply that diabetes has no substantial impact on the excretion of toxic heavy metals in their breast milk. The protein in breast milk is known to have a high potential for heavy metal binding [24]. Mature milk has less protein than Colostrum but more fat and sugar, and the high sugar levels start to decrease at approximately 18 months after birth [25]. It thus implies that as human breast milk matures into the 5–6 weeks after childbirth when fewer proteins and immunoglobulins are expressed in breast milk, the amount of heavy metals bound to the protein expressed in breast milk thus reduces back to near-normal levels [3]. In line with this, similarities in the level of toxic heavy metals in the breast milk of diabetic postpartum mothers should be similar to those of non-diabetic postpartum mothers.

A notable limitation to this study is that it did not assess maternal involvement in clandestine refining of crude oil, illegal mininng and agriculture which involves direct contact with possible toxicants in water and soil. It was omitted from the proforma for data gathering because of ethical issues, as clandestine crude oil refining and illegal minimg are considered social crimes as artisanal refineries and quarries are neither registered nor licensed by the government. The participants were foreseen as not willing to provide such information for fear of arrest and litigation. Given that this study focused on the most harmful (toxic) heavy metals, the Institutional Review Board approved protocol for this study did not include measuring the blood levels of calcium and iron. Low blood Calcium and Iron levels may have resulted in the high Lead and Cadmium levels in breastmilk. This was considered a limitation. Additionally, this study utilized the purposive sampling method which did not offer an equal chance of selection to all members of the target population. The mentioned limitations impose a fair chance of committing type II error. Until further risk assessment studies with bigger samples become available, the generalization of these findings outside of this study group should be done with caution.

### Conclusion

The study participants had an undesirably high concentration of toxic heavy metals in their breast milk above WHO permissible limits. However, our study did not find significant differences in amount of heavy metals excreted in the breast milk of diabetic and non-diabetic postpartum mothers. More toxicological research on mother and child health may be necessary to validate the conclusions of this study.
